# Predictors of one-year mortality at hospital discharge after acute coronary syndromes: A new risk score from the EPICOR (long-tErm follow uP of antithrombotic management patterns In acute CORonary syndrome patients) study

**DOI:** 10.1177/2048872614554198

**Published:** 2015-12

**Authors:** Stuart Pocock, Héctor Bueno, Muriel Licour, Jesús Medina, Lin Zhang, Lieven Annemans, Nicholas Danchin, Yong Huo, Frans Van de Werf

**Affiliations:** 1 London School of Hygiene and Tropical Medicine, UK; 2 Hospital General Universitario Gregorio Marañón, Spain; 3 Medical Department, AstraZeneca France, France; 4 AstraZeneca Observational Research Center, Spain; 5 AstraZeneca Medical Evidence Center, USA; 6 Interuniversity Centre for Health Economics Research UGent, Vrije Universiteit Brussel, Belgium; 7 Hôpital Européen Georges Pompidou, René Descartes University, France; 8 Peking University First Hospital, China; 9 University Hospitals Leuven, Belgium

**Keywords:** Acute coronary syndrome, hospital discharge, mortality, prognostic model, risk score

## Abstract

**Aims:**

A reliable prediction tool is needed to identify acute coronary syndrome (ACS) patients with high mortality risk after their initial hospitalization.

**Methods:**

EPICOR (long-tErm follow uP of antithrombotic management patterns In acute CORonary syndrome patients: NCT01171404) is a prospective cohort study of 10,568 consecutive hospital survivors after an ACS event (4943 ST-segment elevation myocardial infarction (STEMI) and 5625 non-ST-elevation ACS (NSTE-ACS)). Of these cases, 65.1% underwent percutaneous coronary intervention (PCI) and 2.5% coronary artery bypass graft (CABG). Post-discharge mortality was recorded for up to two years. From over 50 potential predictor variables a new risk score for one-year mortality was developed using forward stepwise Cox regression, and examined for goodness-of-fit, discriminatory power, and external validation.

**Results:**

A total of 407 patients (3.9%) died within one year of discharge. We identified 12 highly significant independent predictors of mortality (in order of predictive strength): age, lower ejection fraction, poorer EQ-5D quality of life, elevated serum creatinine, in-hospital cardiac complications, chronic obstructive pulmonary disease, elevated blood glucose, male gender, no PCI/CABG after NSTE-ACS, low hemoglobin, peripheral artery disease, on diuretics at discharge. When combined into a new risk score excellent discrimination was achieved (*c*-statistic=0.81) and this was also validated on a large similar cohort (9907 patients) in Asia (*c*=0.78). For both STEMI and NSTE-ACS there was a steep gradient in one-year mortality ranging from 0.5% in the lowest quintile to 18.2% in the highest decile. NSTE-ACS contributes over twice as many high-risk patients as STEMI.

**Conclusions:**

Post-discharge mortality for ACS patients remains of concern. Our new user-friendly risk score available on www.acsrisk.org can readily identify who is at high risk.

## Introduction

Secondary prevention following an acute coronary syndrome (ACS) event is key as further ischemic events are common following the index event. Risk prediction tools have identified a number of factors which impact on risk of death and myocardial infarction (MI) following an ACS event. However, patient prognosis at hospital discharge continues to vary markedly, and post-discharge mortality remains a concern.^1^ Most risk scores include hospital mortality in their estimations.^2–4^ There are no tools for risk calculation of one-year mortality in hospital survivors. It is usually at the time of discharge that patients are asking about their prognosis. Therefore, there is a need for a reliable prediction tool to identify patients with high mortality risk, which may ultimately allow tailored treatment decisions and improve prognosis. For instance, patients identified as at high risk may receive more frequent follow-up visits to facilitate their optimal care.

For patients experiencing an acute coronary event, a crucial time to assess their prognosis and future management is at discharge from hospital. Hence, there is merit in developing a risk model that utilizes all the patient data on demographics, medical history, and patient status at, and during, admission, and at discharge. From a large representative international cohort study of consecutive patients with ACS who survived to discharge, we have related such detailed patient records to their subsequent follow-up for one year, expressing prognosis in terms of one-year mortality.

While ST segment elevation myocardial infarction (STEMI) and non-ST-elevation ACS (NSTE-ACS) patients have very different management and prognosis patterns during the in-hospital phase, from the moment of hospital discharge there is sufficient common ground and similarity of the key risk factors to combine both sets of patients into a single overall risk model.

There is an extensive literature on risk scores in ACS,^5^ and their use is advocated by the European Society of Cardiology (ESC) guidelines for the management of NSTE-ACS.^6^ However, relatively little attention has been paid to risk assessment at hospital discharge, with just one previous risk score to date regarding six-month mortality post discharge.^7^ This is a valuable opportunity to quantify individual patient risk of mortality to one year after discharge following an acute coronary event hospitalization.

## Methods

EPICOR (long-tErm follow uP of antithrombotic management patterns In acute CORonary syndrome patients) is a prospective, international, observational, real-world practice, cohort study (NCT01171404) comprising consecutive patients, hospitalized for ACS within 24 h of symptom onset, who survived to hospital discharge.

In total, 10,568 patients with non-fatal ACS who survived until hospital discharge were enrolled between September 2010–March 2011 from 555 hospitals in 20 countries across Europe and Latin America. A detailed account of the methodology of the study is described elsewhere.^8,9^

For external validation of our risk model, we used data from the EPICOR-Asia study^10^ (NCT01361386) which enrolled 12,993 patients from eight Asian countries from June 2011–April 2012. This Asian study has followed an almost identical protocol and case record forms as in our EPICOR study.

### Statistical methods

We identified over 50 candidate variables for prediction (patient history, at admission, during admission, and at discharge), and these are listed in Appendix 1. From these a new risk score for one-year mortality post discharge was developed using Cox proportional hazard models. The statistical approach for model building was forward stepwise variable selection, with a criterion of *p*<0.01 for variable inclusion. For continuous predictors, checks were undertaken for non-linearity and, if found appropriate, re-modelling of such variables was conducted e.g. either using a binary cut-off (e.g. hemoglobin, blood glucose) or by expressing as a linear trend above a certain level (e.g. serum creatinine). In combining predictors for patients with STEMI and with NSTE-ACS it is important to explore evidence of statistical interactions with other predictors. On the whole, most variables selected showed a similar magnitude of risk prediction for both STEMI and NSTE-ACS patients. The one exception was that the increased risk of not receiving coronary revascularization during hospitalization was more marked in NSTE-ACS patients.

Some prognostic variables were missing in a small minority of patients. To overcome this problem, thereby enabling all patients’ available data to be validly used, a multiple imputation method was used based on a recently developed extension of the chained equations approach.^11^

Most predictor variables identified (see Table 1) are well understood, but the novel use of the EuroQoL EQ-5D requires explanation. This questionnaire evaluates five issues: patient mobility, self-care, usual activities, pain/discomfort and anxiety/depression. For each there is specific wording to elicit whether the patient has no, moderate, or severe limitation. For each we have scored 0, 1 or 2 points respectively, and adding up these scores yields a simple overall score ranging from 0 points up to a maximum of 10 points. While there do exist more complex weighted schemes for handling the EQ-5D,^12^ we feel that for practical use in our context of user-friendly risk prediction this required the adoption of such simple scoring.

**Table 1.** Descriptive statistics for key baseline variables.

	STEMI patients	NSTE-ACS patients	All	Deaths
No. of patients	4943	5625	10,568	3.9%
STEMI			4943	3.1%
NSTE-ACS			5625	4.5%
Age, years, mean (SD)	59.4 (12.1)	63.8 (12.1)	61.8 (12.3)	
Gender				
Male	3924	3996	7920	3.7%
Female	1019	1629	2648	4.3%
Ejection fraction at admission^a^				
Normal ≥40%	4035	4641	8676	2.9%
Moderately reduced 30–39%	459	329	788	9.0%
Severely reduced <30%	112	126	238	22.7%
Cardiac complications in hospital				
MI or recurrent ischemia	258	342	600	6.7%
Cardiogenic shock	85	24	109	7.3%
Heart failure	327	289	616	12.8%
Any arrhythmia	589	425	1014	6.4%
Any of the above	1019	915	1934	7.6%
Serum creatinine at admission,^a^ mg/dl, mean (SD)	0.96 (0.42)	1.04 (0.59)	1.00 (0.52)	
≥1.2 mg/dl	650	1060	1710	8.9%
High blood glucose (≥160 mg/dl) at admission^a^	1134	1035	2169	6.0%
Low hemoglobin (<13 g/dl) at admission^a^	891	1328	2219	6.9%
COPD or other chronic lung disease	256	427	683	8.8%
Peripheral vascular disease	145	384	529	11.0%
On diuretics at discharge	683	1283	1966	8.5%
Interventions during admission				
CABG or PCI	3863	3285	7148	2.6%
Neither	1080	2340	3420	7.1%
Simple EQ-5D score at discharge^a^				
0, no problems	2392	2382	4774	2.4%
1	1049	1157	2206	3.2%
2	576	785	1361	4.4%
3	335	485	820	4.7%
4	211	325	536	8.4%
≥5, severe problems	226	345	571	11.9%
Geographic region				
Northern Europe	1608	2174	3782	2.5%
Southern Europe	1124	1213	2337	3.6%
Eastern Europe	1145	1235	2380	4.8%
Latin America	1066	1003	2069	5.5%

CABG: coronary artery bypass graft; COPD: chronic obstructive pulmonary disease; MI: myocardial infarction; NSTE-ACS: non-ST-elevation ACS; PCI: percutaneous coronary intervention; SD: standard deviation; STEMI: ST-segment elevation myocardial infarction.

a Indicates variables with missing data as follows: ejection fraction (8.2% missing), serum creatinine (5.6%), blood glucose (13.2%), hemoglobin (6.7%), EQ-5D (2.8%). Multiple imputation was used to overcome this: see statistical methods section.

The multiple imputations were performed using Stata 12.0 while all other analyses used SAS version 9.2.

## Results

The study cohort comprises 10,568 consecutive hospital survivors after an ACS event (4943 STEMI and 5625 NSTE-ACS). Four hundred and seven patients (3.9%) died within one year of discharge while 242 (2.3%) were lost to follow-up.

From all of the candidate variables available, a Cox proportional hazard model was used with forward stepwise variable selection to identify 12 highly significant independent predictors of one-year mortality. These are described in Table 1 along with geographic region.

Table 2 presents the multivariable predictive model which simultaneously uses all 12 risk variables to produce an overall risk score. Variables in Table 2 are listed in order of their statistical significance (age is the strongest predictor) and each hazard ratio is adjusted for all the other variables. One statistical interaction was identified: for NSTE-ACS patients only, those who received percutaneous coronary intervention (PCI) or coronary artery bypass graft (CABG) during this admission had a lower mortality than those on medication only. For continuous variables, potential non-linearity in the prediction of survival was explored. Hence the increasing impact of serum creatinine on mortality was confined to values above 1.2 mg/dl while for blood glucose and hemoglobin binary cut-offs of ≥160 mg/dl and <13 g/dl were respectively used.

**Table 2.** Multivariate analysis of one-year mortality: final model for all patients (with missing data imputed).

Variable	All patients			
	Coefficient	HR	95% CI	*p*-value
Age (per 10 years)	0.43	1.54	1.40–1.70	<0.00001
Ejection fraction <40%^a^	0.62	1.87	1.42–2.46	<0.0001
Ejection fraction <30%^a^	1.35	3.84	2.80–5.27	<0.0001
EQ-5D score (per unit)	0.15	1.16	1.10–1.21	<0.0001
Serum creatinine (per unit ≥1.2 mg/dl)^a^	0.22	1.25	1.13–1.38	<0.0001
Cardiac complication in hospital	0.41	1.50	1.21–1.86	0.0002
Blood glucose ≥160 mg/dl^a^	0.39	1.48	1.19–1.84	0.0004
COPD	0.52	1.68	1.26–2.24	0.0004
Male gender	0.40	1.49	1.18–1.89	0.0009
NSTE-ACS with meds only^b^	0.39	1.47	1.17–1.86	0.0012
NSTE-ACS with PCI/CABG^b^	−0.22	0.80	0.61–1.05	0.1117
Hemoglobin <13 g/dl^a^	0.35	1.42	1.13–1.80	0.0029
Peripheral vascular disease	0.45	1.57	1.17–2.10	0.0029
On diuretics at discharge	0.30	1.35	1.08–1.70	0.0095

CABG: coronary artery bypass graft; CI: confidence interval; COPD: chronic obstructive pulmonary disease; HR: hazard ratio; NSTE-ACS: non-ST-elevation ACS; PCI: percutaneous coronary intervention; STEMI: ST-segment elevation myocardial infarction.

a At admission; ^b^as compared to STEMI.

**Figure 1.**
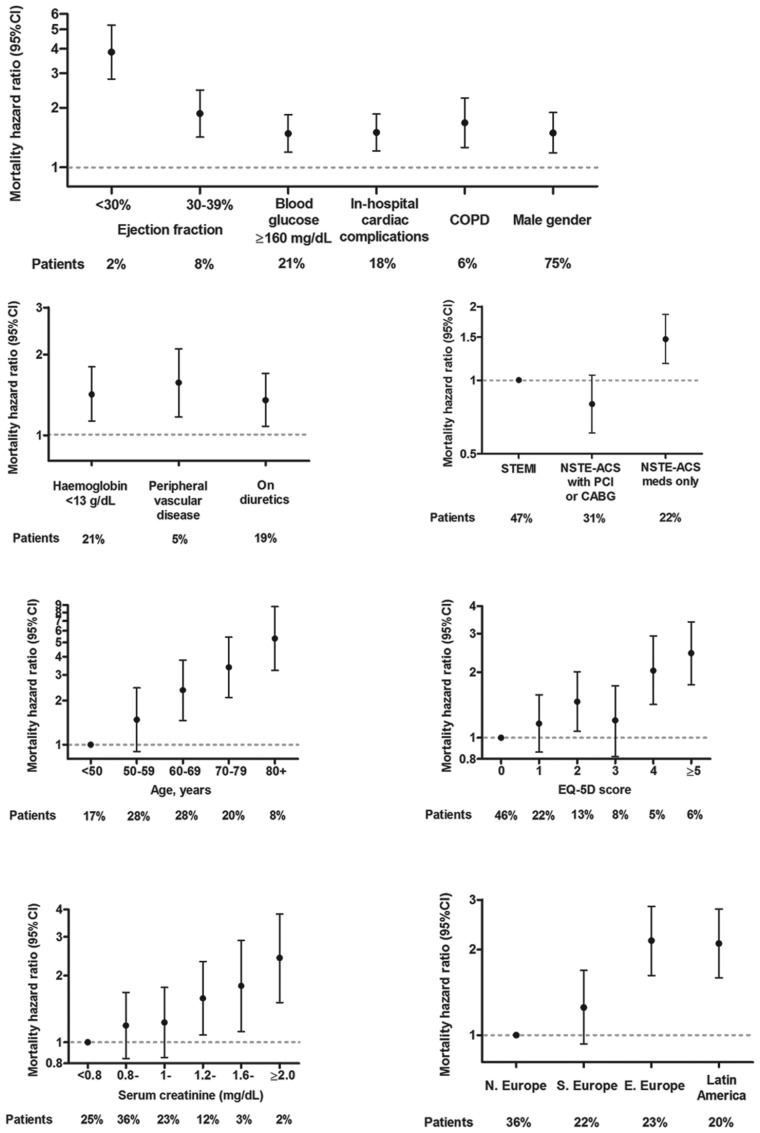
Mortality hazard ratios for each variable in the predictive model. CABG: coronary artery bypass graft; CI: confidence interval; COPD: chronic obstructive pulmonary disease; NSTE-ACS: non-ST-elevation acute coronary syndrome; PCI: percutaneous coronary intervention; STEMI: ST segment elevation myocardial infarction.

Figure 1 displays the independent impact of each predictor on mortality risk. In addition, there remain substantial regional differences in one-year mortality not explained by these predictors: Eastern Europe and Latin American have adjusted hazard ratios of 2.15 and 2.10, respectively, compared with Western Europe (North).

**Figure 2.**
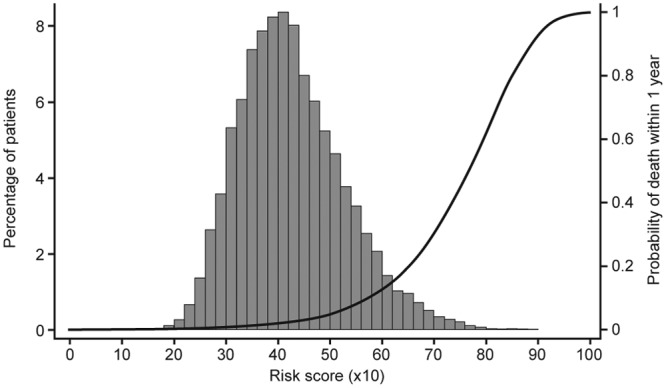
Risk score distribution (and predicted mortality risk).

From the risk coefficients in Table 2, the multivariable risk score is readily calculated for each patient and its distribution (×10) is shown in Figure 2. The curve in Figure 2 relates a patient’s score to the probability of dying within one year of discharge. Good discrimination is achieved with *c*-statistic=0.81.

**Figure 3.**
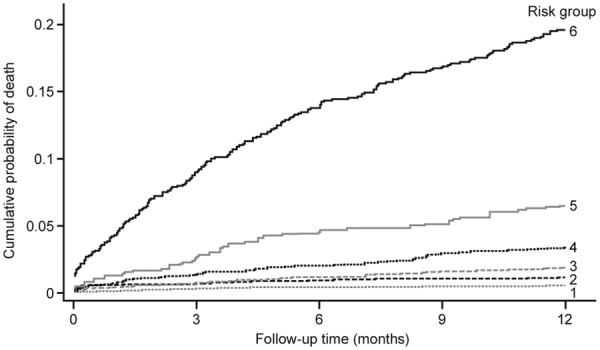
Cumulative mortality in six risk groups. Risk groups 1–4 correspond to quintiles 1–4, with the fifth quintile subdivided into two deciles (risk groups 5 and 6).

Figure 3 shows the cumulative mortality over one year for patients classified into six risk groups. Groups 1–4 comprise the bottom four quintiles of risk while groups 5 and 6 are the top two deciles of risk. While all six groups are clearly separated, the absolute magnitude of differences between risk groups is much more marked for the top two deciles, with 6.3% and 18.2% one-year mortality, respectively. This contrasts with 0.5% one-year mortality in the lowest quintile.

**Figure 4.**
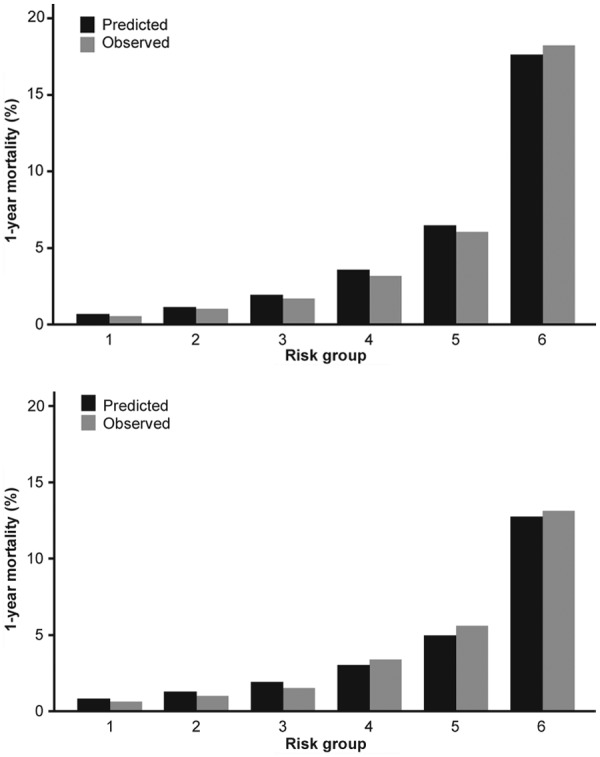
Assessment of risk discrimination and model goodness-of-fit in six groups from low to very high risk (a) In original EPICOR (long-tErm follow uP of antithrombotic management patterns In acute CORonary syndrome patients) study and (b) In EPICOR Asia (validation cohort). For both plots, risk groups 1–4 correspond to quintiles 1–4, with the fifth quintile subdivided into two deciles (risk groups 5 and 6).

Regarding model goodness-of-fit, Figure 4(a) compares observed and model-predicted one-year mortality risk across the six risk groups.

Table 3 shows two separate models for STEMI and NSTE-ACS patients. For nearly all predictors, the strength of mortality association is similar in both subgroups. However, the lower risk if coronary revascularization occurred during admission is more notable in NSTE-ACS patients, and this statistical interaction is captured in the main predictive model in Table 2.

**Table 3.** Separate models for ST-segment elevation myocardial infarction (STEMI) and non-ST-elevation acute coronary syndrome (NSTE-ACS) patients.

Variable	STEMI patients		NSTE–ACS patients	
	HR	95% CI	HR	95% CI
Age (per 10 years)	1.56	1.34–1.80	1.52	1.34–1.73
Ejection fraction <40%^a^	1.42	0.89–2.27	2.29	1.62–3.24
Ejection fraction <30%^a^	3.73	2.17–6.41	4.03	2.69–6.02
EQ-5D score (per unit)	1.18	1.09–1.28	1.14	1.07–1.22
Serum creatinine (per unit ≥1.2 mg/dl)^a^	1.27	1.04–1.55	1.23	1.09–1.38
Cardiac complication in hospital	1.15	0.80–1.65	1.73	1.33–2.27
Blood glucose ≥160 mg/dl^a^	1.29	0.91–1.84	1.64	1.24–2.16
COPD	1.60	0.96–2.68	1.71	1.20–2.43
Male gender	1.47	0.98–2.22	1.54	1.14–2.06
PCI/CABG during admission	0.73	0.52–1.04	0.52	0.40–0.69
Hemoglobin <13 g/dl^a^	1.57	1.05–2.35	1.33	1.00–1.78
Peripheral vascular disease	1.47	0.76–2.86	1.55	1.10–2.18
On diuretics at discharge	1.43	0.97–2.11	1.29	0.97–1.73

CABG: coronary artery bypass graft; CI: confidence interval; COPD: chronic obstructive pulmonary disease; HR: hazard ratio; PCI: percutaneous coronary intervention. ^a^At admission.

For STEMI patients we investigated the impact of rapid time to admission (or time to reperfusion) on reducing mortality after discharge. There were 697 STEMI patients (14%) admitted within one hour of symptom onset: hazard ratio 0.44 (*p*=0.026) compared to other STEMI patients. Also, 1316 STEMI patients (27%) had reperfusion within two hours of symptom onset: hazard ratio 0.64 (*p*=0.053) compared to other STEMI patients. These findings were of borderline statistical significance so these two variables were not included in the main predictive model.

In order to validate our main model on an external cohort, we used the 9907 patients in the EPICOR Asia registry who had complete data on all variables listed in Table 2, of whom 3.1% died within one year of hospital discharge. Figure 4(b) compares the observed and model-predicted mortality in six risk groups (from lowest quintile to top decile). The model fit and extent of risk discrimination is very similar to what was found in our original cohort. The *c*-statistic in EPICOR Asia patients is 0.784, only slightly less than the *c*=0.81 achieved in model development.

## Discussion

The findings we present are based on a large international prospective real-world cohort study comprising consecutive patients hospitalized from an ACS event within 24 h of symptoms onset who survived to hospital discharge. Such a representative population across Europe and Latin America is therefore uniquely well placed to quantify the independent determinants of mortality risk over one year post-discharge.

The 12 highly significant predictors we identified should all be readily available in routine clinical practice. To facilitate the quantification of individual risk we provide a web calculator www.acsrisk.org thus avoiding the burden of numerical calculations.

There is a marked identifiable variation in individual patient risk (see Figure 4). This means a sizeable proportion of patients can be classified as low risk, e.g. around half have a one-year mortality risk <1%. On the other hand 10% of patients have a high one-year mortality risk (see Figures 2 and 3). Knowing this fact, based on our risk model, should help in supporting appropriate patient management.

The contributions made by each specific predictor are worth noting. Not surprisingly, age has the most profound influence on mortality risk, followed by reduced ejection fraction. A more novel contributor to risk assessment is quality of life at discharge, using a simple score derived from the EuroQoL EQ-5D.^13^ Across five aspects (mobility, self-care, usual activities, pain/discomfort, anxiety/discomfort) we add one point for moderate impairment or two points for severe impairment. Patients scoring four points or more (11%) had more than double the mortality risk of patients with no impairment (45%), with a gradient of risk for patients in between these two extremes. Thus, a poor functional quality of life may be expressing some level of frailty and a mortality risk that is not captured by other predictors.

The 4943 STEMI patients had a lower one-year mortality after discharge compared to the 5625 NSTE-ACS patients: 3.1% vs 4.5% died, respectively. However, after adjustment for the other 11 risk factors, the hazard ratio became 1.00 (95% CI 0.80–1.24). This reflects that NSTE-ACS had a higher prevalence of other risk factors (see Table 1). Indeed, NSTE-ACS contributes over twice as many patients in the top decile of risk compared to STEMI. However, for NSTE-ACS patients only, one notable contributor to risk was not having PCI or CABG during hospital stay: hazard ratio 1.84 after adjustment for other risk factors. Thus, lack of coronary revascularization reflects an anticipated poorer prognosis post-discharge. This may be explained by either the actual risk benefit of coronary revascularization or selection bias (i.e. poor risk patients are deemed not appropriate for intervention).^14^

From blood samples at admission, contributions to higher risk are represented by raised creatinine, raised glucose and lower hemoglobin. For disease history, both chronic obstructive pulmonary disease and peripheral vascular disease increased risk, indicating that conditions other than cardiac disease carry a mortality risk. Cardiac complications during the admission were associated with a 50% increase in mortality risk. Also, men had a 50% higher risk than women after all other risk factors were accounted for. In univariate analysis women have a higher one-year mortality than men (4.3% vs 3.7%). But women are more prone to having other risk factors (e.g. older age) so that in the multivariable model being female independently predicts a lower risk. Lastly, being on diuretics at discharge was an indicator of 35% higher mortality risk.

Confidence in the generalizability of any new risk model is much enhanced if it is validated on an external population. Here, the EPICOR Asia study has provided similar risk discrimination and goodness of fit (compare the two plots in Figure 4), as summarized by the *c*-statistic of 0.78 in Asian patients compared to 0.81 in the original cohort. Given that the two studies were from different geographic regions, this provides assurance that our risk model may well be of global applicability.

In external validation some reduction in *c*-statistic is always to be expected on statistical grounds i.e. risk coefficients in any model are optimized by the maximum likelihood principle of any model fit, so the true strength of prediction is inevitably slightly less in another independent data set. To further explore model fit in the Asian cohort we did another Cox regression model with the same 12 predictor variables: the hazard ratios of all but one variable were very similar to those reported in our original EPICOR model (Table 2). The one exception was peripheral vascular disease which was very uncommon in the Asian cohort so that its hazard ratio had a wide CI. This consistency of findings suggests that there is little effect of ethnic diversity on risk prediction.

While there exist several other risk scores for patients with ACS, most do not focus on risk from the time of hospital discharge and hence are not appropriate for comparison here. However, Eagle et al. have used the Global Registry of Acute Coronary Events (GRACE) registry to estimate mortality risk six months post discharge.^7^ Their risk calculator includes nine predictors: age, history of congestive heart failure, history of MI, increased heart rate at admission, lower systolic blood pressure at admission, elevated serum creatinine at admission, elevated cardiac enzymes, ST-segment depression, and no in-hospital PCI. This achieved a similar predictive strength to the current model (*c*-statistic = 0.81 at development, 0.75 at validation). However, the shorter time period could be a limiting factor. Their six-month mortality rate (4.8%) is higher than our one-year mortality rate (3.9%), perhaps reflecting the fact that their cohort is from around 10 years ago. Also, the mortality rate in EPICOR might not include high-risk patients transferred to other units for non-cardiac complications or needing longer-term care. It would be useful if the GRACE and EPICOR risk models were directly compared in an independent cohort of ACS patients followed from hospital discharge.

There are some limitations inherent to our risk model. Being a study on hospital survivors, blood pressure and heart rate at admission were not recorded in our database and hence could not be included in the model. Certain other variables (e.g. probrain natiuretic peptide, incomplete revascularization) were also not available for inclusion. Even after taking account of our 12 highly predictive risk factors, there persist substantial unexplained geographic differences in post-discharge mortality risk. In Eastern Europe and Latin America one-year mortality is markedly higher than in Western Europe, and further investigation is needed to clarify why this discrepancy exists.

This geographic heterogeneity could be perceived as a limitation but given the intention of any risk model is that it be useful in many different countries we feel our population’s geographic diversity is an asset in enhancing generalizability. We intend to publish further on the geographic regional differences in the distribution (prevalence) of risk factors, both in the EPICOR and EPICOR-Asia cohorts.

One could argue that STEMI and NSTE-ACS are sufficiently different conditions that two separate risk models should be developed. However, as shown in Table 3 there is substantial consistency of risk prediction for the 12 variables (i.e. mostly similar hazard ratios) so that for practical purposes we feel a single overall risk model is desirable.

Another limitation is that with over 50 candidate predictor variables there is a risk of a “false positive” predictor entering the risk model. However with *p*<0.01 as entry criterion this risk is relatively low. The one novel predictor is the EQ-5D score, but this is very highly significant. The rest are to be expected on the basis of prior studies of mortality risk in ACS patients.

While the design of EPICOR was geared to recruiting representative patients from representative centers in each country, we cannot directly verify that centers are indeed representative in respect to adherence to treatment guidelines and other aspects of patient management. Thus some caution is warranted with respect to extrapolation of findings to the overall population of ACS patients.

In conclusion, we have documented how post-discharge mortality risk after an ACS event varies markedly. Individual one-year mortality can be reliably estimated using 12 readily available items, and the consequent risk discrimination and model fit are good. User-friendly access to our risk model is available via the web: www.acsrisk.org. We feel this tool can help influence appropriate patient management post discharge, especially in identifying individuals at higher risk for whom more intensive monitoring may be appropriate.

## Appendix

**Appendix 1.** A list of candidate predictor variables.

Demographics and medical history	Variables collected during admission
Gender	Time from symptom onset to admission
Age	Time from admission to reperfusion
Race	Time from symptom onset to reperfusion
Education level	Length of hospital stay
Professional status	Killip class
Height	Diagnosis (STEMI, NSTEMI, unstable angina)
Weight	Left bundle branch block
Body mass index	Ejection fraction^a^
Hypertension	White blood count^a^
Hypercholesterolemia	Creatinine^a^
Diabetes	Glucose^a^
Family history of CAD	Hemoglobin^a^
Smoking	PCI during admission
Previous MI	CABG during admission
Prior PCI	Reperfusion (PCI at thrombolysis)
Prior CABG	No. of dilated vessels
Chronic angina	Any drug eluting stent
Prior heart failure	No. of antiplatelets^b^
Prior atrial fibrillation	Anticoagulant^b^
Prior transient ischemic attack/stroke	Beta blocker^b^
Prior peripheral vascular disease	Angiotensin-converting enzyme inhibitor/angiotensin receptor blocker^b^
Chronic renal failure	Diuretics^b^
COPD or other chronic lung disease	Aldosterone inhibitor^b^
	Calcium-channel blocker^b^
	Ischemic complications
	Cardiogenic shock
	Heart failure
	Dyspnea
	Arrhythmia
	Dependence at discharge
	EQ-5D overall health state at discharge
	EQ-5D simple score at discharge

COPD: chronic obstructive pulmonary disease;

a At admission; ^b^at discharge.
